# 2,6-Diphenyl-3-(prop-2-en-1-yl)piperidin-4-one

**DOI:** 10.1107/S241431462000526X

**Published:** 2020-04-21

**Authors:** V. Manjula, R. Venkateswaramoorthi, J. Dharmaraja, S. Selvanayagam

**Affiliations:** a Department of Chemistry, Periyar University, Salem 636 011, India; b Department of Chemistry, PGP College of Arts and Science, Namakkal 637 207, India; c Department of Chemistry, Anna Government Arts College, Vadachennimalai, Attur 636 121, India; d PG & Research Department of Physics, Government Arts College, Melur 625 106, India; University of Aberdeen, Scotland

**Keywords:** crystal structure, piperidine derivative, C—H⋯π inter­actions

## Abstract

In the title compound, C_20_H_21_NO, the dihedral angle between the phenyl ring is 47.5 (1)° and the piperidine ring adopts a chair conformation. In the crystal, mol­ecules are linked by C—H⋯π inter­actions into dimers with the mol­ecules related by twofold symmetry.

## Abstract 2

In the title piperidine derivative, the mol­ecules are linked by C—H⋯π inter­actions into dimers related by twofold symmetry.

## Graphical abstract

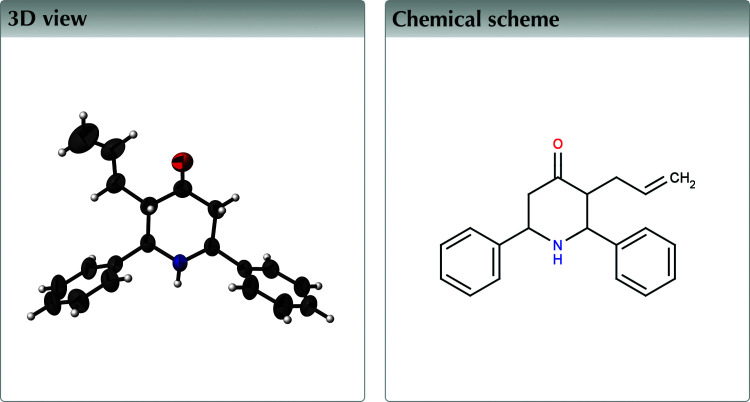

## Structure description

**Figure 1:**
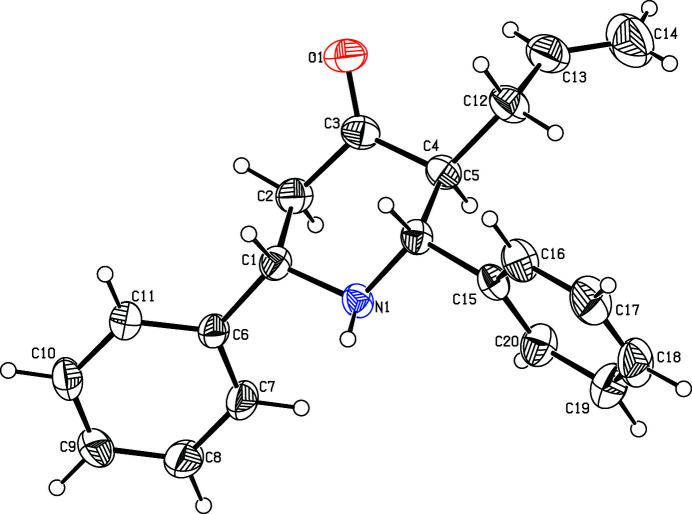
The mol­ecular structure of the title compound, with the atom labelling. Displacement ellipsoids are drawn at the 30% probability level.

Piperidine derivatives can act as enzyme stabilizers to improve therapeutic enzyme activity in Fabry patient cell lines (Li *et al.*, 2018 ▸). Some of these derivatives possess anti­oxidant (Kim *et al.*, 2016 ▸) and analgesic activities (Jahan *et al.*, 2016 ▸). As part of our studies in this area, we now describe the synthesis and structure of the title compound (Fig. 1 ▸).

**Figure 2:**
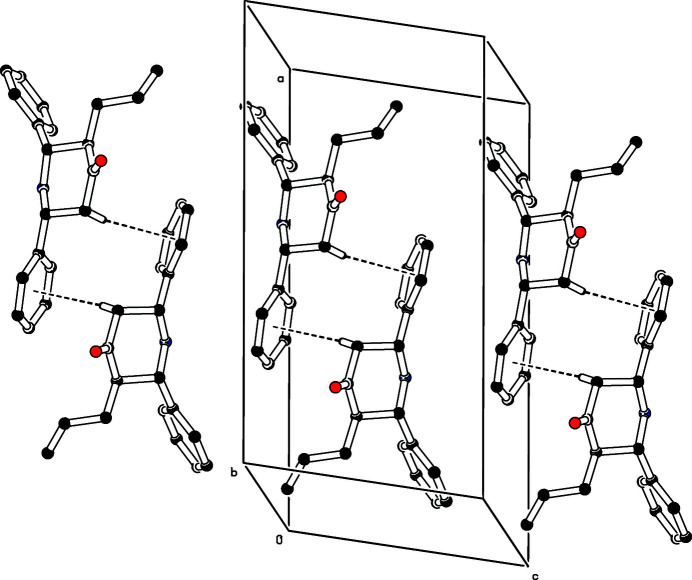
The crystal packing of the title compound viewed along the *b*-axis direction. The C—H⋯π inter­actions are shown as dashed lines. For clarity H atoms not involved in these hydrogen bonds have been omitted.

The piperidine ring adopts a chair conformation and each substituent adopts an equatorial disposition. The dihedral angles between the piperidine ring (all atoms) and the C6–C11 and C15–C20 benzene rings are 70.31 (11) and 79.00 (11)°, respectively. The dihedral angle between the C6–C11 and C15–C20 benzene rings is 47.51 (12)°. In the crystal, a C2—H2⋯*Cg* ^i^ [*Cg* = is the centroid of the C6–C11 ring; symmetry code: (i) 1 − *x*, *y*, 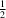 − *z*) inter­action occurs with H2*A*⋯*Cg* = 2.73 Å and C2—H2*A*⋯*Cg* = 148°. This leads to dimers with the mol­ecules related by twofold rotation symmetry (Fig. 2 ▸). The N1—H1*N* grouping points towards the opposite face of the C6–C11 ring but the H1*N*⋯*Cg* separation of 3.15 Å is probably too long to be regarded as a bond.

## Synthesis and crystallization

A mixture of hexene-2-one (0.05 mol), benzaldehyde (0.1 mol), ammonium acetate (0.05 mol) and ethanol (40 ml) was heated gently and poured into ether (50 ml) and treated with concentrated hydro­chloric acid (25 ml). The precipitated hydro­chloride was washed with an ethanol–ether mixture. The base was liberated by adding strong ammonia until the hydro­chloride dissolved. Dilution with water afforded the free base. The pure compound was was further recrystallized with benzene–petroleum ether to yield the title compound.

## Refinement

Crystal data, data collection and structure refinement details are summarized in Table 1 ▸.

## Supplementary Material

Crystal structure: contains datablock(s) I, global. DOI: 10.1107/S241431462000526X/hb4345sup1.cif

Structure factors: contains datablock(s) I. DOI: 10.1107/S241431462000526X/hb4345Isup2.hkl

CCDC reference: 1996936

Additional supporting information:  crystallographic information; 3D view; checkCIF report

## Figures and Tables

**Table 1:** Experimental details

Crystal data	
Chemical formula	C_20_H_21_NO
*M*_r_	291.38
Crystal system, space group	Monoclinic, *C*2/*c*
Temperature (K)	296
*a*, *b*, *c* (Å)	16.7588 (4), 23.4597 (5), 8.7769 (2)
β (°)	98.771 (1)
*V* (Å^3^)	3410.34 (13)
*Z*	8
Radiation type	Mo *K*α
μ (mm^−1^)	0.07
Crystal size (mm)	0.24 × 0.21 × 0.19

Data collection	
Diffractometer	Bruker SMART APEX CCD
No. of measured, independent and observed [*I* > 2σ(*I*)] reflections	28337, 5208, 2586
*R*_int_	0.071
(sin θ/λ)_max_ (Å^−1^)	0.714

Refinement	
*R*[*F* ^2^ > 2σ(*F* ^2^)], *wR*(*F* ^2^), *S*	0.080, 0.201, 1.03
No. of reflections	5208
No. of parameters	203
No. of restraints	1
H-atom treatment	H atoms treated by a mixture of independent and constrained refinement
Δρ_max_, Δρ_min_ (e Å^−3^)	0.22, −0.19

Computer programs: *SMART* and *SAINT* (Bruker, 2002 ▸), *SHELXS97* (Sheldrick, 2008 ▸), *SHELXL2018/3* (Sheldrick, 2015 ▸), *ORTEP-3 for Windows* (Farrugia, 2012 ▸) and *PLATON* (Spek, 2020 ▸).

## full crystallographic data

### Crystal data

**Table:**

C_20_H_21_NO	*F*(000) = 1248
*M**_r_* = 291.38	*D*_x_ = 1.135 Mg m^−^^3^
Monoclinic, *C*2/*c*	Mo *K*α radiation, λ = 0.71073 Å
*a* = 16.7588 (4) Å	Cell parameters from 12118 reflections
*b* = 23.4597 (5) Å	θ = 2.6–25.8°
*c* = 8.7769 (2) Å	µ = 0.07 mm^−^^1^
β = 98.771 (1)°	*T* = 296 K
*V* = 3410.34 (13) Å^3^	Block, colourless
*Z* = 8	0.24 × 0.21 × 0.19 mm

### Data collection

**Table:**

Bruker SMART APEX CCD diffractometer	*R*_int_ = 0.071
Radiation source: fine-focus sealed tube	θ_max_ = 30.5°, θ_min_ = 2.5°
ω and φ scans	*h* = −23→23
28337 measured reflections	*k* = −24→33
5208 independent reflections	*l* = −12→12
2586 reflections with *I* > 2σ(*I*)	

### Refinement

**Table:**

Refinement on *F*^2^	Primary atom site location: structure-invariant direct methods
Least-squares matrix: full	Hydrogen site location: mixed
*R*[*F*^2^ > 2σ(*F*^2^)] = 0.080	H atoms treated by a mixture of independent and constrained refinement
*wR*(*F*^2^) = 0.201	*w* = 1/[σ^2^(*F*_o_^2^) + (0.0736*P*)^2^ + 2.1792*P*] where *P* = (*F*_o_^2^ + 2*F*_c_^2^)/3
*S* = 1.03	(Δ/σ)_max_ < 0.001
5208 reflections	Δρ_max_ = 0.22 e Å^−^^3^
203 parameters	Δρ_min_ = −0.19 e Å^−^^3^
1 restraint	

### Special details

**Table:**

Geometry. All esds (except the esd in the dihedral angle between two l.s. planes) are estimated using the full covariance matrix. The cell esds are taken into account individually in the estimation of esds in distances, angles and torsion angles; correlations between esds in cell parameters are only used when they are defined by crystal symmetry. An approximate (isotropic) treatment of cell esds is used for estimating esds involving l.s. planes.
Refinement. The C-bound H atoms were placed in idealized locations and refined as riding atoms. The N-bound H atom was located in a difference map and refined with a restraint.

### Fractional atomic coordinates and isotropic or equivalent isotropic displacement parameters (Å^2^)

**Table:**

	*x*	*y*	*z*	*U*_iso_*/*U*_eq_	
H1N	0.1365 (10)	0.3964 (7)	0.4008 (13)	0.034 (5)*	
O1	0.20727 (10)	0.25243 (7)	0.7596 (2)	0.0756 (5)	
N1	0.14621 (9)	0.38387 (7)	0.4932 (2)	0.0440 (4)	
C1	0.08589 (11)	0.34078 (9)	0.5144 (2)	0.0454 (5)	
H1	0.091163	0.308720	0.445077	0.054*	
C2	0.10339 (12)	0.31960 (10)	0.6811 (3)	0.0537 (5)	
H2A	0.093153	0.349981	0.750586	0.064*	
H2B	0.067787	0.288036	0.694966	0.064*	
C3	0.18939 (13)	0.30065 (10)	0.7189 (2)	0.0520 (5)	
C4	0.25116 (12)	0.34443 (9)	0.6888 (2)	0.0475 (5)	
H4	0.244557	0.378105	0.751803	0.057*	
C5	0.22884 (11)	0.36147 (9)	0.5167 (2)	0.0437 (5)	
H5	0.230394	0.327262	0.453108	0.052*	
C6	0.00096 (11)	0.36388 (9)	0.4799 (2)	0.0457 (5)	
C7	−0.01719 (13)	0.41865 (10)	0.5187 (3)	0.0700 (7)	
H7	0.023993	0.443022	0.561372	0.084*	
C8	−0.09651 (15)	0.43787 (11)	0.4947 (4)	0.0828 (9)	
H8	−0.108226	0.474984	0.521265	0.099*	
C9	−0.15798 (13)	0.40190 (12)	0.4315 (3)	0.0697 (7)	
H9	−0.210973	0.414956	0.413381	0.084*	
C10	−0.14082 (13)	0.34762 (12)	0.3960 (3)	0.0604 (6)	
H10	−0.182316	0.323007	0.356199	0.072*	
C11	−0.06178 (12)	0.32856 (10)	0.4187 (2)	0.0516 (5)	
H11	−0.050688	0.291315	0.392277	0.062*	
C12	0.33802 (13)	0.32300 (11)	0.7319 (3)	0.0639 (6)	
H12A	0.342670	0.286037	0.684469	0.077*	
H12B	0.374059	0.349014	0.689870	0.077*	
C13	0.36444 (17)	0.31778 (12)	0.9013 (3)	0.0778 (8)	
H13	0.330510	0.298403	0.958095	0.093*	
C14	0.4310 (2)	0.33817 (16)	0.9754 (5)	0.1174 (13)	
H14A	0.466503	0.357808	0.922576	0.141*	
H14B	0.443395	0.333178	1.081520	0.141*	
C15	0.28595 (11)	0.40515 (9)	0.4675 (2)	0.0456 (5)	
C16	0.34151 (12)	0.38922 (11)	0.3724 (3)	0.0612 (6)	
H16	0.342811	0.351815	0.337808	0.073*	
C17	0.39434 (14)	0.42874 (14)	0.3297 (3)	0.0784 (8)	
H17	0.431583	0.417627	0.267007	0.094*	
C18	0.39317 (15)	0.48407 (15)	0.3776 (3)	0.0789 (8)	
H18	0.429663	0.510311	0.348618	0.095*	
C19	0.33762 (16)	0.50067 (12)	0.4689 (3)	0.0748 (7)	
H19	0.335531	0.538466	0.500032	0.090*	
C20	0.28487 (13)	0.46112 (10)	0.5145 (3)	0.0609 (6)	
H20	0.248064	0.472486	0.577783	0.073*	

### Atomic displacement parameters (Å^2^)

**Table:**

	*U*^11^	*U*^22^	*U*^33^	*U*^12^	*U*^13^	*U*^23^
O1	0.0690 (11)	0.0525 (11)	0.1045 (14)	0.0095 (8)	0.0110 (9)	0.0197 (10)
N1	0.0348 (8)	0.0518 (11)	0.0463 (10)	−0.0014 (7)	0.0090 (7)	0.0037 (8)
C1	0.0413 (10)	0.0423 (12)	0.0544 (12)	−0.0013 (8)	0.0133 (9)	−0.0044 (9)
C2	0.0476 (12)	0.0546 (13)	0.0624 (14)	0.0022 (10)	0.0193 (10)	0.0084 (11)
C3	0.0529 (12)	0.0532 (14)	0.0512 (12)	0.0091 (10)	0.0125 (9)	0.0024 (10)
C4	0.0439 (11)	0.0518 (12)	0.0478 (11)	0.0071 (9)	0.0098 (9)	−0.0018 (9)
C5	0.0374 (10)	0.0478 (12)	0.0479 (11)	0.0005 (8)	0.0128 (8)	−0.0060 (9)
C6	0.0385 (10)	0.0456 (12)	0.0544 (12)	−0.0045 (8)	0.0119 (9)	0.0031 (9)
C7	0.0404 (12)	0.0480 (14)	0.122 (2)	−0.0064 (10)	0.0126 (12)	−0.0035 (14)
C8	0.0521 (14)	0.0491 (15)	0.148 (3)	0.0066 (11)	0.0168 (15)	0.0025 (16)
C9	0.0395 (12)	0.0760 (18)	0.0925 (19)	0.0035 (11)	0.0060 (12)	0.0143 (14)
C10	0.0430 (12)	0.0775 (18)	0.0586 (14)	−0.0155 (11)	0.0011 (10)	0.0021 (12)
C11	0.0498 (12)	0.0539 (13)	0.0513 (12)	−0.0096 (10)	0.0079 (9)	−0.0038 (10)
C12	0.0488 (13)	0.0726 (16)	0.0698 (16)	0.0117 (11)	0.0069 (11)	0.0055 (12)
C13	0.0720 (17)	0.0709 (18)	0.0851 (19)	0.0158 (14)	−0.0048 (14)	−0.0004 (14)
C14	0.105 (3)	0.120 (3)	0.113 (3)	0.007 (2)	−0.029 (2)	−0.028 (2)
C15	0.0353 (10)	0.0602 (14)	0.0413 (10)	0.0017 (9)	0.0059 (8)	−0.0004 (9)
C16	0.0417 (12)	0.0793 (17)	0.0663 (14)	0.0030 (11)	0.0197 (10)	−0.0045 (12)
C17	0.0470 (13)	0.108 (2)	0.0869 (19)	−0.0006 (14)	0.0309 (13)	0.0039 (17)
C18	0.0528 (14)	0.103 (2)	0.0825 (18)	−0.0246 (15)	0.0156 (13)	0.0143 (17)
C19	0.0742 (17)	0.0693 (18)	0.0820 (18)	−0.0205 (13)	0.0160 (14)	−0.0002 (14)
C20	0.0563 (13)	0.0646 (16)	0.0657 (15)	−0.0075 (11)	0.0221 (11)	−0.0078 (12)

### Geometric parameters (Å, º)

**Table:**

O1—C3	1.210 (3)	C9—H9	0.9300
N1—C1	1.461 (2)	C10—C11	1.383 (3)
N1—C5	1.466 (2)	C10—H10	0.9300
N1—H1N	0.854 (9)	C11—H11	0.9300
C1—C6	1.510 (3)	C12—C13	1.491 (4)
C1—C2	1.531 (3)	C12—H12A	0.9700
C1—H1	0.9800	C12—H12B	0.9700
C2—C3	1.496 (3)	C13—C14	1.295 (4)
C2—H2A	0.9700	C13—H13	0.9300
C2—H2B	0.9700	C14—H14A	0.9300
C3—C4	1.510 (3)	C14—H14B	0.9300
C4—C12	1.532 (3)	C15—C20	1.377 (3)
C4—C5	1.552 (3)	C15—C16	1.393 (3)
C4—H4	0.9800	C16—C17	1.373 (4)
C5—C15	1.509 (3)	C16—H16	0.9300
C5—H5	0.9800	C17—C18	1.365 (4)
C6—C7	1.375 (3)	C17—H17	0.9300
C6—C11	1.381 (3)	C18—C19	1.374 (4)
C7—C8	1.389 (3)	C18—H18	0.9300
C7—H7	0.9300	C19—C20	1.382 (3)
C8—C9	1.381 (4)	C19—H19	0.9300
C8—H8	0.9300	C20—H20	0.9300
C9—C10	1.352 (3)		
			
C1—N1—C5	113.02 (16)	C10—C9—C8	119.8 (2)
C1—N1—H1N	108.8 (12)	C10—C9—H9	120.1
C5—N1—H1N	107.2 (12)	C8—C9—H9	120.1
N1—C1—C6	112.05 (16)	C9—C10—C11	120.2 (2)
N1—C1—C2	108.11 (16)	C9—C10—H10	119.9
C6—C1—C2	110.23 (16)	C11—C10—H10	119.9
N1—C1—H1	108.8	C6—C11—C10	121.2 (2)
C6—C1—H1	108.8	C6—C11—H11	119.4
C2—C1—H1	108.8	C10—C11—H11	119.4
C3—C2—C1	110.24 (16)	C13—C12—C4	113.5 (2)
C3—C2—H2A	109.6	C13—C12—H12A	108.9
C1—C2—H2A	109.6	C4—C12—H12A	108.9
C3—C2—H2B	109.6	C13—C12—H12B	108.9
C1—C2—H2B	109.6	C4—C12—H12B	108.9
H2A—C2—H2B	108.1	H12A—C12—H12B	107.7
O1—C3—C2	122.0 (2)	C14—C13—C12	125.0 (3)
O1—C3—C4	122.82 (19)	C14—C13—H13	117.5
C2—C3—C4	114.97 (18)	C12—C13—H13	117.5
C3—C4—C12	112.63 (19)	C13—C14—H14A	120.0
C3—C4—C5	105.99 (17)	C13—C14—H14B	120.0
C12—C4—C5	113.78 (16)	H14A—C14—H14B	120.0
C3—C4—H4	108.1	C20—C15—C16	118.4 (2)
C12—C4—H4	108.1	C20—C15—C5	121.60 (18)
C5—C4—H4	108.1	C16—C15—C5	120.0 (2)
N1—C5—C15	110.06 (16)	C17—C16—C15	120.0 (2)
N1—C5—C4	108.14 (14)	C17—C16—H16	120.0
C15—C5—C4	112.57 (16)	C15—C16—H16	120.0
N1—C5—H5	108.7	C18—C17—C16	121.2 (2)
C15—C5—H5	108.7	C18—C17—H17	119.4
C4—C5—H5	108.7	C16—C17—H17	119.4
C7—C6—C11	118.21 (19)	C17—C18—C19	119.5 (2)
C7—C6—C1	121.52 (18)	C17—C18—H18	120.3
C11—C6—C1	120.08 (19)	C19—C18—H18	120.3
C6—C7—C8	120.6 (2)	C18—C19—C20	119.9 (3)
C6—C7—H7	119.7	C18—C19—H19	120.0
C8—C7—H7	119.7	C20—C19—H19	120.0
C9—C8—C7	120.0 (2)	C15—C20—C19	121.0 (2)
C9—C8—H8	120.0	C15—C20—H20	119.5
C7—C8—H8	120.0	C19—C20—H20	119.5
			
C5—N1—C1—C6	−176.38 (16)	C6—C7—C8—C9	0.0 (5)
C5—N1—C1—C2	61.9 (2)	C7—C8—C9—C10	1.3 (4)
N1—C1—C2—C3	−53.1 (2)	C8—C9—C10—C11	−1.9 (4)
C6—C1—C2—C3	−175.88 (17)	C7—C6—C11—C10	0.4 (3)
C1—C2—C3—O1	−120.6 (2)	C1—C6—C11—C10	175.5 (2)
C1—C2—C3—C4	54.2 (2)	C9—C10—C11—C6	1.0 (3)
O1—C3—C4—C12	−6.1 (3)	C3—C4—C12—C13	−70.9 (3)
C2—C3—C4—C12	179.14 (18)	C5—C4—C12—C13	168.4 (2)
O1—C3—C4—C5	118.9 (2)	C4—C12—C13—C14	−130.5 (3)
C2—C3—C4—C5	−55.8 (2)	N1—C5—C15—C20	48.1 (2)
C1—N1—C5—C15	170.62 (16)	C4—C5—C15—C20	−72.7 (2)
C1—N1—C5—C4	−66.1 (2)	N1—C5—C15—C16	−131.9 (2)
C3—C4—C5—N1	58.6 (2)	C4—C5—C15—C16	107.4 (2)
C12—C4—C5—N1	−177.06 (18)	C20—C15—C16—C17	1.1 (3)
C3—C4—C5—C15	−179.56 (16)	C5—C15—C16—C17	−179.0 (2)
C12—C4—C5—C15	−55.3 (2)	C15—C16—C17—C18	−0.7 (4)
N1—C1—C6—C7	−37.4 (3)	C16—C17—C18—C19	−0.6 (4)
C2—C1—C6—C7	83.0 (3)	C17—C18—C19—C20	1.5 (4)
N1—C1—C6—C11	147.64 (19)	C16—C15—C20—C19	−0.2 (3)
C2—C1—C6—C11	−91.9 (2)	C5—C15—C20—C19	179.8 (2)
C11—C6—C7—C8	−0.9 (4)	C18—C19—C20—C15	−1.1 (4)
C1—C6—C7—C8	−175.9 (2)		
